# Elucidating the Role of *Toxoplasma gondii*’s Mitochondrial Superoxide Dismutase

**DOI:** 10.3390/biom15070972

**Published:** 2025-07-07

**Authors:** James Alexander Tirtorahardjo, Christopher I-H. Ma, Areej Shaikh, Rosa M. Andrade

**Affiliations:** 1 Department of Cell and Developmental Biology, University of California San Diego, La Jolla, CA 92093, USA; jtirtorahardjo@ucsd.edu; 2 Department of Medicine, Division of Infectious Diseases, University of California Irvine, Irvine, CA 92697, USA; arshaikh@uci.edu; 3 Department of Microbiology and Molecular Genetics, University of California Irvine, Irvine, CA 92697, USA

**Keywords:** Toxoplasma, mitochondria, superoxide dismutase, electron transport chain, ATP synthase

## Abstract

*Toxoplasma gondii* is an Apicomplexan parasite that possesses a well-developed system of scavengers of reactive oxygen species (ROS). Among its components, *T. gondii* mitochondrial superoxide dismutase (TgSOD2) is essential, as predicted by the CRISPR phenotype index and evidenced by the non-viability of its constitutive knockouts. As an obligate intracellular parasite, TgSOD2 is upregulated during extracellular stages. Herein, we generated a viable TgSOD2 knockdown mutant using an inducible auxin–degron system to explore the biological role of TgSOD2 in *T. gondii*. Depletion of TgSOD2 led to impaired parasite growth and replication, reduced mitochondrial membrane potential (MMP), abnormalities in the distribution of ATP synthase within its mitochondrial electron transport chain (mETC), and increased susceptibility to mETC inhibitors. Through a proximal biotinylation approach, we identified the interactions of TgSOD2 with complexes IV and V of its mETC, suggesting that these sites are sensitive to ROS. Our study provides the first insights into the role of TgSOD2 in maintaining its mitochondrial redox homeostasis and subsequent parasite replication fitness. **Significance:** *Toxoplasma gondii* infects nearly a third of the world population and can cause fetal miscarriages or life-threatening complications in vulnerable patients. Current therapies do not eradicate the parasite from the human hosts, rendering them at risk of recurrence during their lifetimes. *T. gondii* has a single mitochondrion, which is well-known for its susceptibility to oxidative damage that leads to *T. gondii*’s death. Therefore, targeting *T. gondii* mitochondrion remains an attractive therapeutic strategy for drug development. *T. gondii*’s mitochondrial superoxide dismutase is an antioxidant protein in the parasite mitochondrion and is essential for its survival. Understanding its biological role could reveal mitochondrial vulnerabilities in *T. gondii* and provide new leads for the development of effective treatments for *T. gondii* infections.

## 1. Introduction

*Toxoplasma gondii*, a human pathogen that infects nearly a third of the world’s population [1], is a significant global health concern. It is vulnerable to endogenous reactive oxygen species (ROS) and those generated by the host cell. To survive, *T. gondii* has evolved a highly developed and redundant ROS scavenging system, allowing it to infect a wide range of nucleated host cells, including phagocytic cells capable of ROS bursts [2].

Notably, *T. gondii* encodes three distinct superoxide dismutases (SOD) within its ROS scavenging system: a cytoplasmic TgSOD1 [3], TgSOD2 in its mitochondrial matrix [4], and TgSOD3 expressed in the mitochondrion of *T. gondii* oocysts [5]. The latter is only expressed when oocysts are formed in the definitive feline host.

Superoxide dismutases are the only enzymes responsible for the dismutation of superoxide anions into hydrogen peroxide. In *T. gondii*, TgSOD1 and TgSOD2 are critical for its survival *in vitro*, as demonstrated by Odberg-Ferragut et al. when they generated non-viable knockout parasites [3]. Since then, no further attempts have been made to explore the roles of superoxide dismutases in *T. gondii* biology. CRISPR/CAS9 screens of these genes suggest that all three SODs are highly fitness-conferring [6].

*T. gondii* possesses a single mitochondrion, an organelle readily affected by oxidative stress and nutritional deficiencies, which is critical for *T. gondii* survival [7,8]. Within its mitochondrion, TgSOD2 localizes to the matrix. However, little is known about the contribution of TgSOD2 to *T. gondii* mitochondrial biology and homeostasis. Understanding the biological role of TgSOD2 may help discover parasite vulnerabilities that can be further mined for drug development.

Our study examines the role of TgSOD2 and demonstrates how its depletion impairs parasite replication, mitochondrial membrane potential (MMP), and leads to abnormalities in its mitochondrial electron transport chain (mETC). These data suggest that TgSOD2 plays an important role in maintaining *T. gondii* mitochondrial homeostasis. Our research on TgSOD2 could contribute to the development of new strategies to combat this widespread and potentially deadly infection.

## 2. Materials and Methods

### 2.1. Host Cell and Parasite Cultures

All parasite strains used in this study were derived from Type I RH. They were grown and maintained in human foreskin fibroblasts (HFF) in Dulbecco’s modified Eagle’s medium supplemented with 10% fetal bovine serum (Hyclone), penicillin and streptomycin (50 mg/mL each), and 200 µM of L-glutamine and maintained via serial passage at 37 °C and 5% CO_2_. For passage, extracellular parasites were collected following lysis from HFF cells and introduced into new, confluent monolayers of HFF every 48 h. Each passage involved 100 µL of inoculum of tachyzoites. Mini auxin-inducible degron (mAID) parasites were maintained in complete media containing dialyzed fetal bovine serum (Gibco #A3382001). Parasites and their origin are listed in Table S1.

### 2.2. Transcription Quantification of ROS Scavenger Genes in T. gondii Parasites

For intracellular parasites, a confluent monolayer infected with parasites was used to isolate the parasites and extract their RNA immediately after. For extracellular parasites, a confluent monolayer infected with parasites was mechanically lysed to synchronize extracellular time. These extracellular parasites were incubated in a host cell-free environment for 1 or 4 h before RNA extraction. RNA extraction was performed with TRizol (Invitrogen), purified with an RNeasy Mini Kit (Qiagen), and cDNA synthesis was carried out with an iScript cDNA Synthesis Kit (Bio-Rad), followed by qPCR performed with an iTaq Universal SYBR Green Supermix (Bio-Rad). Gene IDs and primers are listed in Table S2. Relative quantification was determined using the comparative threshold cycle (ΔΔCT) method. Results from three independent experiments are presented.

### 2.3. Generation of Mutant Parasites

Mutants were generated using CAS9-directed homologous recombination. Guide RNA (gRNA) was selected based on the Eukaryotic Pathogen CRISPR guide RNA/DNA Design Tool (http://grna.ctegd.uga.edu/) [9] targeting the 3′ end of TgSOD2 (primers in Table S3) and inserted into a pU6 plasmid containing both CAS9 and a gRNA expression cassette. The resulting plasmid was used to transform *E. coli* for plasmid amplification. Additionally, homologous recombination tags along with an HXGPRT expression cassette for xanthine and mycophenolic acid selection were generated on their corresponding plasmids (Table S4). These homologous recombination inserts were purified before use in transfection and dilutional cloning. The expression of target tags was confirmed via PCR, target genomic sequencing, and fluorescence microscopy.

### 2.4. Parasite Transfection

Parasites were pelleted at 400× *g* for 10 min at 18 °C and resuspended with Cytomix buffer (10 mM KPO_4_, 120 mM KCl, 5 mM MgCl_2_, 25 mM HEPES, 2 mM EDTA, 2 µM of ATP, and 5 µM of glutathione) and combined with DNA to a final volume of 400 µL. Parasites were electroporated using an ECM 630 Electro Cell Manipulator (BTX) in 4 mm cuvettes with the following settings: at 2000 V, 25 ohm resistance, and 50 µF capacitance. Resulting transfectant pools were subjected to dilutional cloning. Tagging was confirmed via PCR, genomic sequencing, and fluorescence microscopy.

### 2.5. Western Blotting

Collected parasites were washed 2 timeswith PBS, then resuspended in 100 µL of RIPA buffer supplemented with EDTA-free protease inhibitors (cOmplete™ 11836170001 Roche) and lysed on ice for 30 min. Insoluble cell components were then collected via centrifugation at 10,000× *g* at 4 °C for 10 min. A total of 10 µg of protein was loaded for each lane of the 12% polyacrylamide gel. For Western blotting, the gel was run in TRIS/glycine running buffer (Bio-Rad), transferred onto a nitrocellulose membrane, and blocked in 5% milk powder, 5% goat serum, and 0.1% Tween-20 in Tris-buffered saline. TgSOD2 was identified via mouse anti-HA antibody (BioLegend, clone 16B12). The molecular weight of our experimental tags ranged from 40 kDa to 70 kDa: TgSOD2 tags ~70 kDa and TgApiCOX35 40 kDa. We selected *T. gondii* Dense Granule proteins (GR) as suitable controls due to their lower molecular weight than standard controls such as actin (32 kDa) or GADPH (53 kDa). They are constitutively expressed, making them suitable controls for our Western blotting experiments [10,11,12]. Monoclonal antibodies were obtained using BEI resources, NIAID, NIH: Monoclonal Anti-*Toxoplasma gondii* Dense Granule Antigen 1, Clone T5 2B4 (produced *in vitro*), NR-50264; Monoclonal Anti-*Toxoplasma gondii* Dense Granule Antigen 2, Clone T4 1F5 (produced *in vitro*), NR-50260. Lastly, the blot was stained with anti-mouse HRP-conjugated antibody and visualized using Bio-Rad’s Clarity ECL solution [13,14]. Original figures can be found in supplementary materials.

### 2.6. Parasite Plaque Assay

A 6-well plate with a confluent monolayer of HFFs in complete media with dialyzed serum or supplemented with 500 µM IAA was infected with 100 parasites per well and allowed to incubate undisturbed for 8 days. Then, the 6-well plates were washed with PBS, fixed with methanol, and then stained with crystal violet [15]. Plaques were manually counted, and the area of each identified plaque was quantified via ImageJ version 1.53j [16].

### 2.7. Parasite Replication Assay

A confluent 18 mm coverslip in complete media containing dialyzed serum was infected with 1,000,000 parasites (MOI of 5) and allowed to invade for 1 h. Then, the media were replaced with either complete media or 500µM IAA-containing media, then returned to the incubator for 24 h for replication. For mETC inhibitor treatment, the media also included 5μM of BSO (L-buthionine sulfoximine)—an inducer of ROS that is not toxic to mammalian cells [17]—and either 20 nM antimycin (complex III inhibitor), 0.5 µM oligomycin (ATP synthase/complex V inhibitor), or both. Fluorescent microscopy images were taken, and the number of parasites per vacuole was counted. Vacuoles were tallied as containing 1, 2, 4, 8, 16, or 32 parasites. Images of 20 random fields of view per experimental condition per experiment were quantified in this manner.

### 2.8. Mitochondrial Membrane Potential

TgSOD2-mAID-3HA-YFP parasites were isolated as previously described [18], centrifuged at 400× *g* for 10 min, and incubated with 200 nM MitoTracker Red CMXRos (Life Technologies) in complete media for 25 min in a 37 °C incubator at 5% CO_2_. After staining, parasites were pelleted at 500 g for 5 min and resuspended in PBS to be analyzed via flow cytometry (NovoCyte flow cytometer Agilent) and FlowJo V10 software (Tree Star Inc.).

### 2.9. Mitochondrial Morphology Microscopy

A confluent monolayer of HFF in an 18 mm coverslip was infected with 1,000,000 parasites (MOI of 5). Following 1 h of invasion, the monolayer was washed with PBS and then replaced with either media supplemented with dialyzed serum or 500 µM of IAA-containing media. The monolayer was then returned to the incubator for 24 h to allow for replication. Coverslips were stained with an anti-F1B antibody to label parasite mitochondria. Fluorescent microscopy images were taken, and the mitochondria were classified as intact, aberrant, or absent. Images of 20 random fields per coverslip from three independent experiments, each with three technical replicates, were collected.

## 3. Results

### 3.1. Expression of T. gondii Superoxide Dismutase 1 and 2 Increases in Extracellular Parasites

**Figure 1:**
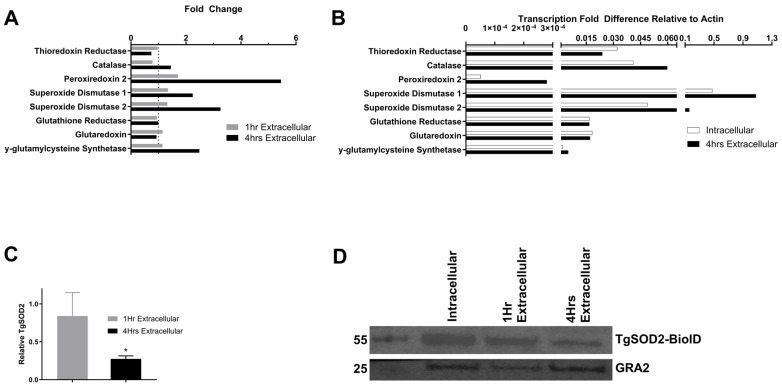
TgSOD1 and TgSOD2 are the most commonly transcribed ROS scavengers during extracellular stress. Intracellular parasites were collected from confluent monolayers of fibroblasts infected with wild-type RH parasites. For extracellular stages, filtered parasites were collected after 1 h and 4 h for RNA extraction and qPCR (primers listed in Table S1). (**A**) Transcription fold difference normalized to actin. (**B**) Average fold changes relative to intracellular parasites. (**C**) Quantification of TgSOD2 in extracellular parasites over time relative to intracellular parasites, after normalization to GRA2 loading control. *n* = 3 independent experiments. * *p* < 0.05. Error bars indicate standard deviation. Comparison tests and graphs were performed using GraphPad Prism version 10.0.0 for Windows, GraphPad Software, Boston, Massachusetts, USA, www.graphpad.com. (**D**) Representative Western blot showing decreasing expression of TgSOD2 over time (GRA2 loading control). The parasite clone with TgSOD2-BioID-3HA was probed with an anti-HA antibody.

TgSOD1 and TgSOD2 were the most transcribed ROS scavengers in both intracellular and extracellular stages of *T. gondii* tachyzoites (Figure 1A). Notably, transcripts of TgSOD1 showed a 2-fold increase, and TgSOD2 increased 3-fold 4 h after exiting the host cells (Figure 1B). Peroxiredoxin 2 was the only other scavenger that showed a significant fold increase in transcripts after 4 h of extracellular stress. However, this gene is considered dispensable for parasite survival [6]. At the translational level, a significant reduction in TgSOD2 protein was observed during extracellular stages, which worsens over time (*p* < 0.05) (Figure 1C,D).

### 3.2. Generation of an Inducible TgSOD2 Knockdown Clone

**Figure 2:**
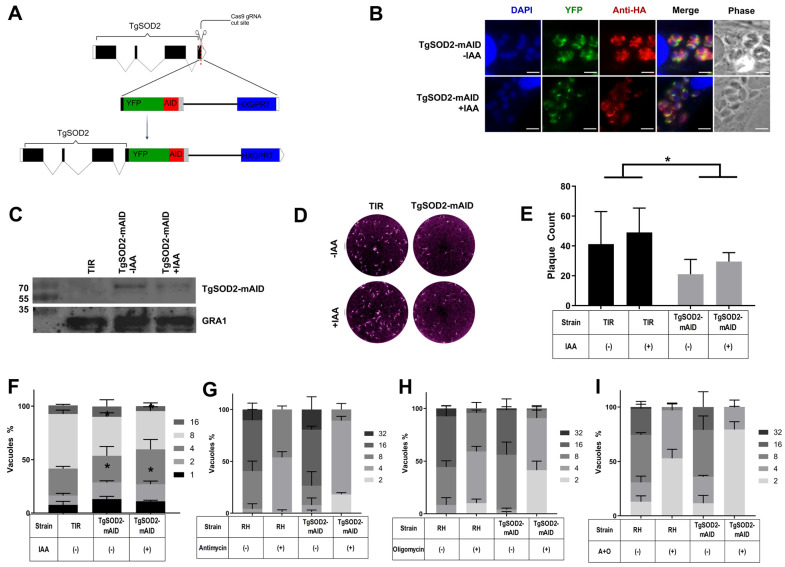
TgSOD2 depletion in TgSOD2-mAID parasites reduced growth and replication. (**A**) Insertion of the YFP-mAID-HXGPRT at 3′ end of TgSOD2 via CRISPR/CAS9. Created with Biorender [22] (**B**) Microscopy validation of YFP fluorescence, HA tags, and mAID-mediated TgSOD2 knockdown after treatment with IAA for 24 h. The scale bars are 5 μm. (**C**) Representative Western blot of TgSOD2 in TgSOD2-BioID and TgSOD2-mAID. Both retained the native SOD2 promoter; however, TgSOD2-mAID is susceptible to proteasomal degradation in the presence of IAA. Stained with anti-HA antibodies, with GRA1 as loading control. TgSOD2-mAID showed near depletion of TgSOD2 after 24 h of IAA exposure. (**D**) Representative plaque assay after 8 days of incubation ±IAA for parental control (TIR) or TgSOD2-mAID parasites. (**E**) Plaque assay quantification following 8 days of incubation ±IAA. (**F**) Replication assay following 24 h of incubation ±IAA. (**G**–**I**) Replication assay following 24 h of IAA exposure and BSO addition for ROS induction. Wild-type RH and TgSOD2-mAID parasites were treated with or without mETC inhibitors antimycin A (complex III inhibitor, (**G**)), oligomycin (ATP synthase/complex V inhibitor, (**H**)), or both (**I**). For all quantifications: *n* = 3 independent experiments. Error bars indicate standard deviation. * *p* < 0.05. Comparison tests and graphs were performed using GraphPad Prism version 10.0.0 for Windows, GraphPad Software, www.graphpad.com.

To directly investigate TgSOD2, an auxin-inducible knockdown strain of wild-type RH parasites was generated. This approach was first successfully applied in *T. gondii* for cytoplasmic proteins [19]. It has also succeeded for proteins localized in other *T. gondii* organelles, such as the apicoplast and the mitochondrion [20]. Using CAS9-directed homologous recombination, a YFP tag and mini auxin-inducible degron (mAID-YFP-3xHA) tag [21] were appended onto the 3′ end of the TgSOD2 gene (Figure 2A). The resulting parasite strain (TgSOD2YFP-mAID) should undergo proteasomal degradation of TgSOD2 upon exposure to indoleacetic acid (IAA). As demonstrated via fluorescent microscopy, TgSOD2YFP-mAID retained its mitochondrial distribution (Figure 2B top row) and showed a decreased expression of TgSOD2YFP upon exposure to IAA for 24 h (Figure 2B bottom row). Given that inducible knockdown systems are known to introduce a degree of baseline target protein knockdown in the absence of the knockdown signal, Western blots were performed to quantitatively compare TgSOD2 levels in a non-knockdown system (TgSOD2-BioID) and those found in our TgSOD2-mAID parasites before and after the induction of proteasomal degradation (with or without IAA). Because TgSOD2-BioID and TgSOD2-mAID both retained the same endogenous promoter for TgSOD2, the expression of TgSOD2 between the two mutants should be similar in the absence of IAA. However, our TgSOD2-mIAD parasites showed significantly reduced levels of TgSOD2 when compared to TgSOD2-BioID parasites. Notably, near depletion of TgSOD2 was achieved after incubation with IAA for 24 h (Figure 2C).

### 3.3. TgSOD2 Depletion Impairs Parasite Growth

Following validation of the auxin-inducible degron mutant, the phenotype of TgSOD2 depletion was assessed. First, plaque assays were performed to determine the overall changes to parasite growth following the depletion of TgSOD2. After 8 days of IAA exposure, the TgSOD2-mAID mutants exhibited a baseline decrease in replication compared to the transport inhibitor response 1 (TIR-1) parental line (Figure 2D). TgSOD2-mAID parasites also formed fewer plaques, demonstrating a reduction in general growth (Figure 2D,E). Infected host cells with TgSOD2-mIAD had fewer parasites per PV (Figure 2F). Neither the number of plaques nor the number of parasites per PV was significantly affected by exposure to IAA. However, the reduction in parasites per PV was more pronounced in TgSOD2-mAID parasites treated with mETC inhibitors (antimycin, oligomycin, or both) (Figure 2G–I), suggesting their increased susceptibility to oxidative stress.

### 3.4. TgSOD2 Depletion Alters the Parasite Mitochondrial Homeostasis

**Figure 3:**
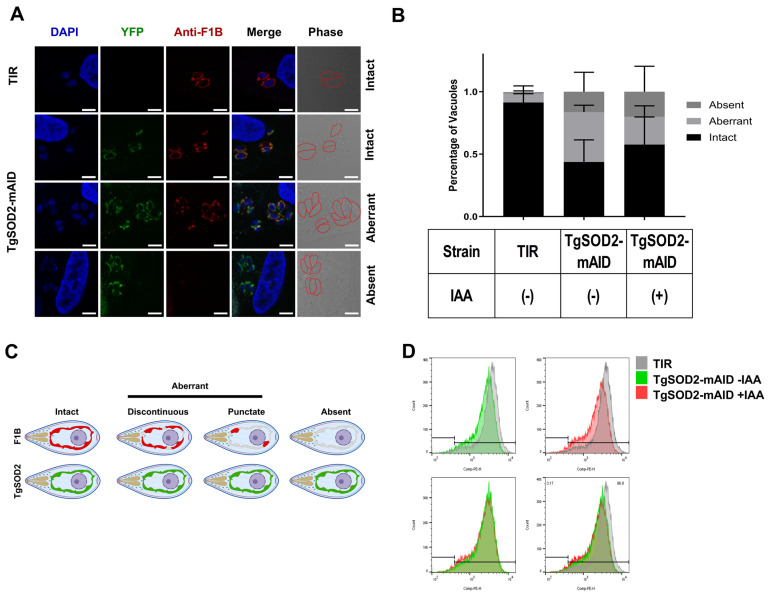
TgSOD2-mIAD parasites have abnormal mitochondrial ATP synthase distribution and reduced mitochondrial membrane potential. (**A**) Parasites stained with anti-F1B ATP synthase antibodies. YFP fluorescence in TgSOD2-mAID did not overlap with ATP synthase staining in parasites. The scale bars are 5 μm. (**B**) Phenotypic scoring of vacuoles with normal or aberrant mitochondria based on the staining patterns of ATP synthase. *n* = 3 independent experiments. Error bars indicate standard deviation. Comparisons tests and graphs were performed using GraphPad Prism version 10.0.0 for Windows, GraphPad Software, Boston, MA, USA, www.graphpad.com. (**C**) Schematic illustration of staining patterns observed for ATPase synthase. The aberrant group includes both discontinuous and punctate staining signals. Created with Biorender [23] (**D**) MMP in TgSOD2-mAID parasites was reduced compared to their parental controls. MMP was measured via MitoTracker staining after 24 h of IAA treatment. Representative flow cytometry histogram shown was developed from 3 independent experiments.

TgSOD2-mAID mutants retained their mitochondrial localization and lasso-like mitochondrial morphology (Figure 2C), as confirmed by the yellow fluorescence signals and staining with an anti-F1B ATP synthase antibody (Figure 3A). However, these mutants showed significant abnormalities in ATP synthase distribution within the parasite mitochondria, as demonstrated by immunofluorescence microscopy. Specifically, ATP synthase in the TgSOD2-mAID mutants did not show spatial overlap with TgSOD2 when compared to their TIR-1 parental line (Figure 3A,B), and its distribution was disrupted in roughly 50% of mutant parasites (Figure 3B,C). In TgSOD2-depleted mutants, ATP synthase either displayed punctate/discontinuous localization or was completely absent. Notably, IAA exposure did not induce further changes.

Since TgSOD2 is a mitochondrial matrix protein and TgSOD2-mAID parasites displayed an aberrant distribution of their mitochondrial ATP synthase, we decided to explore the effect of TgSOD2 knockdown on *T. gondii*’s MMP using a MitoTracker assay measured with flow cytometry. MMP decreased in TgSOD2-mAID mutants compared to the TIR-1 parental line, and this effect in mutants was not further altered with IAA exposure for 24 h (Figure 3D).

### 3.5. Generation of TgSOD2-BioID to Evaluate Protein–Protein Interactions

**Figure 4:**
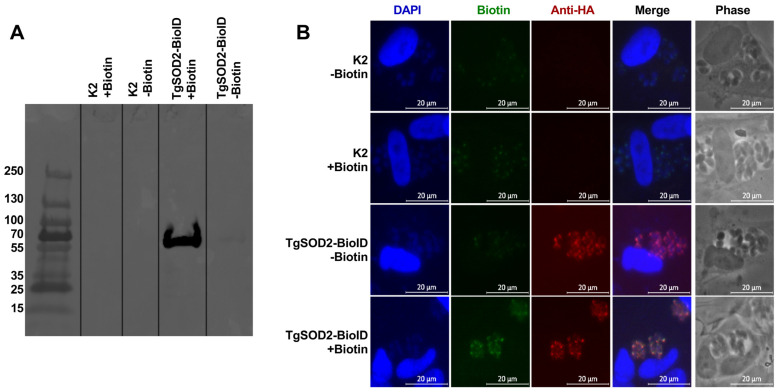
Successful generation of TgSOD2-BioID line: (**A**) Streptavidin pulldown of TgSOD2-BioID or parental (K2) lysates after culturing in biotin-containing media. Western blot showed an expected ~70 kDa band in the TgSOD2-BioID lysates. Lysates probed with α-HA antibody. (**B**) Fluorescence microscopy shows mitochondrial localization of the TgSOD2-BioID fusion protein. TgSOD2 was identified via anti-HA tag staining (red), biotin staining via streptavidin-FITC (green), and nuclear staining via DAPI (blue). The scale bars are 5 μm.

Given the importance of TgSOD2 to *T. gondii* survival and its localization in the mitochondrial matrix, we next explored the interaction between TgSOD2 and other mitochondrial proteins [3,4,24]. The proximal biotin ligation approach was adapted to identify these potential interactions, as previously described [24,25]. Briefly, a TgSOD2-BirA*-3xHA construct retaining its native endogenous TgSOD2 promoter was generated and genomically integrated into ∆Ku80∆HXGPRT RH parasites (parental K2). Western blots were performed to assess the labeling of *T. gondii* SOD2 via TgSOD2-BioID, with the whole-cell lysates probed by an anti-HA antibody conjugated with a streptavidin probe (Figure 4A). This relied upon BioID, a promiscuous biotin ligase, to self-biotinylate when exposed to free biotin. A band of 70 KDa, corresponding to the TgSOD2-BioID, was observed in this Western blot, confirming the activity of BioID in the fusion protein to self-biotinylate (Figure 4A). Additionally, immunofluorescence microscopy confirmed the localization of TgSOD2-BioID in the mitochondria, exhibiting a lasso-like mitochondrial morphology (Figure 4B).

To determine if TgSOD2-BioID could label proximal proteins in the mitochondria, parasites were grown in a medium supplemented with biotin and stained for biotinylated proteins using FITC-conjugated streptavidin. Parental parasites (K2) showed negligible streptavidin labeling (Figure 4B, biotin column, top two rows). In TgSOD2-BioID parasites, robust streptavidin staining with mitochondrial distribution was detected, indicating that the biotin ligase fusion was active and that BioID-labeled proteins were in the mitochondria (Figure 4B, biotin panel, bottom row).

### 3.6. TgSOD2 Is in Close Proximity to Proteins of Its Mitochondrial Electron Transport Chain

With biotin ligase activity confirmed, the next step was biotin-labeled protein capture via streptavidin-conjugated magnetic beads. Mass spectrometry analysis of the streptavidin-captured proteins revealed that TgSOD2-BioID biotin ligation enriched the capture of several proteins of the parasite’s mETC (Table 1): TgApiCox18, TgApiCox26, TgApiCox30, TgApiCox35, and ATP synthase beta subunit 5. The TgApiCox proteins are components of the cytochrome C oxidase (Complex IV), and the ATP synthase beta subunit 5 is part of ATP synthase (Complex V).

To assess whether any of these proximal proteins had strong interactions with TgSOD2, an anti-HA pulldown was performed. Anti-HA beads should capture TgSOD2-BioID and any closely associated proteins in direct contact with the target (TgSOD2). This swap in pulldown methodology should result in a more stringent capture of possible interacting proteins. Only *T. gondii*’s ATP synthase beta subunit 5 was detected (Table 1).

**Figure 5:**
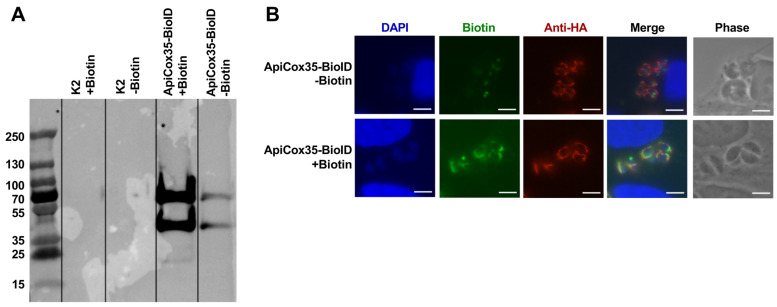
Generation of TgApiCox35-BioID line. (**A**) Streptavidin pulldown of TgApiCox35-BioID or parental (K2) lysates after culture in biotin-containing media. Western blot showed the capture of an expected ~70 kDa band in the TgApiCox35-BioID lysate. (**B**) Fluorescence microscopy shows mitochondrial localization of the TgApiCox35-BioID fusion protein. TgApiCox35 was identified via anti-HA tag staining (red), biotin staining via streptavidin-FITC (green), and nuclear staining via DAPI (blue). The scale bars are 5 μm.

To identify reciprocal protein–protein interactions between TgSOD2 and any of the specific TgApiCOX proteins detected by our streptavidin pulldown, we selected TgApiCox35 as a representative complex IV subunit to generate a TgApiCox35-BioID mutant. Western blot following streptavidin pulldown (Figure 5A) and microscopic verification (Figure 5B) identified TgApiCox35-BioID via HA tagging and biotin ligase activity (staining with YFP-conjugated streptavidin). The TgApiCox35-BioID tag is localized adequately to *T. gondii* mitochondria and is capable of proximal biotin ligation (Figure 5B). With biotin ligase activity confirmed, the next step was to capture and identify biotinylated proteins via mass spectrometry. Mass spectrometry of the streptavidin-captured proteins revealed the enrichment of other TgApiCox proteins (TgApiCox 16, 18, 19, 24, 25, 26, and 30), cytochrome C, and ATP synthase B subunits (Table 2). However, TgSOD2 was absent from this pulldown.

### 3.7. TgApiCox Transcription Patterns Mirror TgSOD2 Transcription

**Figure 6:**
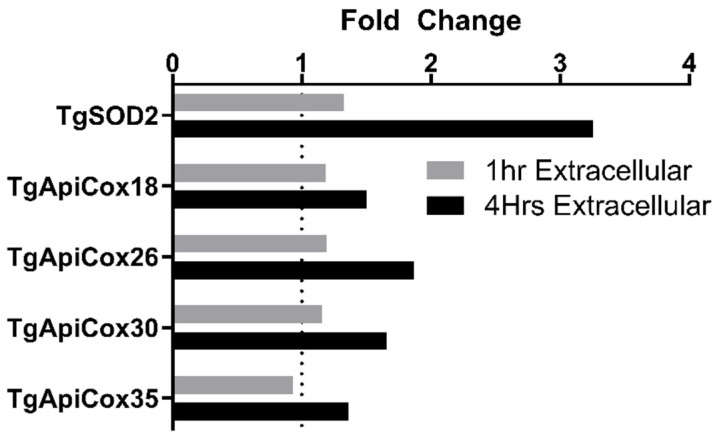
qPCR survey of TgApiCox in intracellular and extracellular parasites. For intracellular parasites, confluent monolayers of fibroblasts infected with wild-type RH parasites were collected after washing with PBS. For extracellular stages, filtered parasites were collected after 1 and 4 h of incubation. At each time point, RNA was harvested for cDNA synthesis and real-time qPCR of *T. gondii* TgApiCox genes. Data normalized to *T. gondii* actin and presented as an average fold change against intracellular parasites. *n* = 3 independent experiments. Comparison tests and graphs were performed using GraphPad Prism version 10.0.0 for Windows, GraphPad Software, Boston, MA, USA, www.graphpad.com.

Previously generated cDNA from wild-type parasites (Section 2.2) was used to evaluate the transcription patterns of the TgApiCox proteins found in close proximity to TgSOD2. Extracellular parasites exhibited increases in TgApiCox18, TgApiCox26, TgApiCox30, and TgApiCox35 transcription after being forcibly egressed from host cells for 4 h. This pattern mirrors the transcriptional response of TgSOD2 to extracellular stress (Figure 6).

## 4. Discussion

We report the first viable *T. gondii* mutant with a significant deficiency in mitochondrial superoxide dismutase (TgSOD2). This mutant enabled us to identify the role of TgSOD2 in *T. gondii* fitness and its mitochondrial membrane potential (MMP)—a substantial reduction in TgSOD2 decreased parasite fitness, reduced MMP, and increased susceptibility to reactive oxygen species (ROS). It also resulted in an abnormal distribution of complex V (ATP synthase) in the parasite’s mitochondria. Since TgSOD2 was found in close proximity to complex V (ATP synthase) of its mETC, TgSOD2 may be relevant for ATP synthase structural maintenance within the parasite mitochondrion. Our findings suggest that TgSOD2 may play a role in protecting *T. gondii*’s mitochondrion.

As expected for an obligate intracellular parasite, the expression of ROS scavengers increases as the parasite egresses the host cells and remains in the extracellular environment, as shown in qPCR assays (Figure 1A,B). Notably, TgSOD2 is highly transcribed in extracellular parasites, representing the parasite’s response to decreasing amounts of TgSOD2 protein (Figure 1C,D). These findings correlate with previous reports comparing transcriptomics before and during host cell invasion [26]. Similar to TgSOD2, the transcription of members of *T. gondii* mETC is upregulated during extracellular stress (Figure 6).

Previous attempts to knock out TgSOD2 rendered the parasites non-viable, indicating that TgSOD2 is essential for parasite survival [3]. Herein, we successfully generated the knockdown of TgSOD2 using an auxin-inducible degron system via CRISPR/CAS9 (Figure 2A). This methodology introduces a smaller insertion, limiting disruptions to other proximal genes. This has been previously applied in *T. gondii* for proteins in the cytoplasm [19], apicoplast, and mitochondria of the parasite [20]. We selected this auxin-degron system given its known rapid degradation of cytoplasmic proteins upon exposure to auxin (within seconds or minutes) [19]. This feature is highly desirable to prevent compensatory mechanisms of survival in inducible knockdown mutants. This contrasts with other, more frequently used approaches, such as tetracycline-inducible systems [27]. Notably, TgSOD2 replication, transcription, and translation start outside the mitochondria before its post-translational delivery into the matrix of the mitochondria due to its signal peptide [2]. Hence, AID tag-mediated depletion of mitochondrial proteins would rely upon degrading nascent protein following cytoplasmic translation [28]. To ensure this phenotype always remains consistent, the mutant parasites were maintained in dialyzed media to minimize unintended knockdown from auxin-like compounds present in standard tissue culture media [29]. These optimizations enabled us to achieve success in depleting TgSOD2 (Figure 2B,C). As with other conditional knockdown systems, some degree of off-target degradation occurs with the AID system for cytoplasmic proteins. To our surprise, our validated TgSOD2-mAID mutant exhibited a remarkably low level of TgSOD2 at baseline, which made any further depletion in the presence of auxin appear insignificant (Figure 2C). Considering that these experiments were performed in the absence of any stressors or at baseline, TgSOD2 degradation may have occurred during each round of replication of the dilutional cloning process. Hence, complementation of our TgSOD2-mAID mutant to restore its wild-type phenotype might not be as straightforward as expected, but will be part of our future studies.

Our resulting parasite, TgSOD2-mAID, was viable but exhibited reduced replication fitness, as observed in our replication and plaque assays (Figure 2D–I). This was expected as TgSOD2 is considered an essential gene for parasite fitness [6]. Exposure to auxin did not further inhibit plaque formation or exacerbate fitness loss in the TgSOD2-mAID mutants, which is consistent with our observations during validation assays. Whether *T. gondii* mitochondria are permeable to auxin or contain a similar ubiquitin–proteasome degradation system as such in its cytoplasm remains to be elucidated.

Our TgSOD2-mAID mutants preserved their mitochondrial localization and distribution (Figure 3A,C). They also exhibited reduced MMP (Figure 3D) and abnormal ATP synthase distribution patterns (Figure 3B,C). Morphological abnormalities in the mitochondria of TgSOD2-mAID parasites, along with a decrease in MMP, suggested that TgSOD2 helped maintain both the structure and function of the parasite mitochondria, which may be indicative of mitochondrial stress and predictive of parasite apoptosis [7]. They could also suggest a link between the depletion of TgSOD2 and the distribution of ATP synthase or the assembly of this multi-subunit complex. These knockdown parasites were also more susceptible to the effects of mETC inhibitors, such as antimycin [30] and oligomycin [31], which dramatically reduced TgSOD2-mAID replication rates (Figure 2H,I). These effects were not associated with their impact on host cells, as we worked with doses of mETC inhibitors that did not affect their viability (unpublished preliminary data). Notably, TgSOD2 depletion led to more severe replication defects when parasites were treated with the complex III inhibitor antimycin [30] (Figure 2G), which might suggest that *T. gondii* complex III is the most ROS-susceptible member of the parasite’s mETC.

To evaluate if TgSOD2 has a role in the homeostasis of *T. gondii* mitochondria, we assessed the interactions between TgSOD2 and its neighboring mitochondrial proteins through a proximal biotinylation approach (Figure 4) wherein biotin ligase labels all proteins within 10 nm, regardless of whether the protein was bound to TgSOD2 [32]. Mass spectrometry of the streptavidin-captured proteins identified members of the electron transport chain complexes, specifically the TgApiCox subunits (complex IV) and ATP synthase β-subunit (complex V) (Table 1). Interestingly, the captured proteins were all predicted to have a CRISPR fitness score of less than -3, indicating that they are highly fitness-conferring [6]. To determine if any of these proteins form strong bonds with TgSOD2, we performed HA-tag pulldown assays with our TgSOD2-BioID mutants, given that they contain an HA-tag. Unlike the previous capture, anti-HA capture relied on proteins forming stronger interactions with TgSOD2-BioID, the only HA-tagged protein in the sample. This more stringent anti-HA pulldown captured ATP synthase β-subunit (complex V) in addition to other mitochondrial proteins not shown in the streptavidin pulldown (Table 1). These findings are in line with previous reports that iron-dependent SODs form homodimers to function; however, no other protein–protein interactions involving TgSODs have been identified [3]. Thus, it is unlikely that TgSOD2 would form covalent bonds with complexes IV or V. Instead, this data suggests that TgSOD2 is found in proximity to those complexes at baseline. TgSOD2 may be a necessary bystander that maintains electron transport chain function by neutralizing superoxide anions generated in the vicinity. Whether these interactions vary depending on *T. gondii* stage or stressors remains unknown.

Since multiple TgApiCox subunits (complex IV) were captured in the streptavidin pulldown, we selected a representative TgApiCox protein (TgApiCox35) to test whether this proximity is reciprocal. We generated and validated a TgApiCox35-BioID mutant (Figure 5). Unlike the TgSOD2-BioID pulldown, TgApiCox35 pulldown captured proteins associated with complex III in addition to complex IV, but not TgSOD2 (Table 2). Notably, our results identified TgApiCox25, which has been previously reported to be in proximity to TgApiCox35 [33]. This lack of reciprocity between TgApiCox35 and TgSOD2 does not rule out the potential role of TgSOD2 as a ROS scavenger in protecting complex IV and its TgApiCox complex subunits.

Our study is the first to suggest a relationship between TgSOD2 and ATP synthase β-subunit. In other organisms, loss of SOD also contributed to decreased ATP production, low membrane potential, and general mitochondrial dysfunction [34]. Given evidence that TgSOD2 does not directly bind to ATP synthase [35], it is most likely that TgSOD2, being a mitochondrial matrix protein, localizes near ROS generation points or near the most ROS-sensitive proteins inside the *T. gondii* mitochondrion. This seems most likely given that Apicomplexan electron transport chains have larger membrane-bound electron transport proteins and more subunits, resulting in higher-molecular-weight complexes [36]. These features may shift ROS generation to different members of the electron transport chain or render different components more vulnerable to ROS-mediated damage.

Apicomplexan parasites possess unique iron-dependent SODs that set them apart from mammalian SODs. Despite their potential in drug discovery, the role of mitochondrial superoxide dismutases in *T. gondii* remained elusive. Thanks to the advent of novel molecular biology techniques, our viable TgSOD2 knockdown mutants offer an opportunity to further explore the *T. gondii* mitochondrion as a therapeutic target and gain a deeper understanding of its susceptibility to oxidative damage, which is essential for the parasite’s survival.

## 5. Conclusions

Our study provides the first direct evidence that TgSOD2 plays a role in *T. gondii*’s mitochondrial redox homeostasis at baseline. Our future directions include the exploration of the role of TgSOD2 on mitochondrial health during stressful conditions, as well as generating type II mutants with TgSOD2 deficiency. Type II parasites mimic clinically relevant strains of *T. gondii*. Overall, understanding *T. gondii* mitochondrial redox homeostasis could reveal *T. gondii* vulnerabilities and provide new leads for the therapeutic development of curative treatments for *T. gondii* infections.

## Figures and Tables

**Table 1:** Mass spectrometry identification of streptavidin-captured proteins from TgSOD2-BioID and parental lines. Liquid chromatography–mass spectrometry analysis used an UltiMate 3000 RSLC system (Thermo Fisher Scientific) coupled in-line to an Orbitrap Fusion Lumos mass spectrometer (Thermo Fisher Scientific). Enriched proteins in the SOD2-BioID sample were identified by aligning them to a Toxoplasma proteome and are shown here.

ToxoDB GeneID	Protein Annotation	Capture Method	Predicted Protein Mass (kDa)	Protein Localization	Phenotype Score (Sidik et al., 2016 [6])	Human Homolog Similarity
TGME49_316330	superoxide dismutase TgSOD2 (SOD2)	Streptavidin Beads	31.47	Mitochondrion	−4.09	38.97%
TGME49_221510	Tg ApiCox18	Streptavidin Beads	17.92	Mitochondrion	−3.28	0.00%
TGME49_306670	Tg ApiCox26	Streptavidin Beads	25.84	Mitochondrion	−3.68	0.00%
TGME49_297810	Tg ApiCox30	Streptavidin Beads	29.57	Mitochondrion	−3.64	0.00%
TGME49_229920	Tg ApiCox35	Streptavidin Beads	34.96	Mitochondrion	−3.84	0.00%
TGME49_261950	ATP synthase beta subunit ATP-B (ATPB)	Streptavidin Beads	59.93	Mitochondrion	−4.84	79.45%
TGME49_209260	cytochrome c oxidase subunit 5b	Streptavidin Beads	34.71	Mitochondrion	−3.07	38.60%
TGME49_260170	elongation factor EF-G, putative	Streptavidin Beads	96.63	Mitochondrion	−4.87	51.36%
TGME49_257730	methionine aminopeptidase, type I, putative	Streptavidin Beads	52.24	Mitochondrion	−3.51	50.00%
TGME49_202680	peptidase M16, alpha subunit, putative	Streptavidin Beads	62.21	Mitochondrion	−4.3	32.96%
TGME49_236210	peptidase M16 family protein, putative	Streptavidin Beads	56.92	Mitochondrion	−4.74	44.15%
TGME49_216530	ribosome recycling factor protein	Streptavidin Beads	51.08	Mitochondrion	−3.81	0.00%
TGME49_316330	superoxide dismutase SOD2 (SOD2)	Anti-HA Beads	31.47	Mitochondrion	−4.09	38.97%
TGME49_261950	ATP synthase beta subunit ATP-B (ATPB)	Anti-HA Beads	59.93	Mitochondrion	−4.84	79.45%
TGME49_247550	heat shock protein HSP60	Anti-HA Beads	60.92	Mitochondrion	−5.1	55.51%
TGME49_288500	FAD Malate-dehydrogenase (MDH-FAD)	Anti-HA Beads	60.39	Mitochondrion	−0.79	0.00%
TGME49_243950	prohibitin, putative	Anti-HA Beads	30.23	Mitochondrion	−5.18	51.49%
TGME49_219550	dihydrolipoyllysine-residue succinyltransferase component of oxoglutarate dehydrogenase	Anti-HA Beads	50.13	Mitochondrion	−4.25	60.26%
TGME49_215590	flavoprotein subunit of succinate dehydrogenase	Anti-HA Beads	72.76	Mitochondrion	−3.96	65.70%
TGME49_319920	2-oxo acid dehydrogenases acyltransferase (catalytic domain) domain-containing protein	Anti-HA Beads	70.3	Mitochondrion	−1.92	40.60%
TGME49_204400	ATPase synthase subunit alpha, putative	Anti-HA Beads	62.16	Mitochondrion	−3.84	74.11%

**Table 2:** Mass spectrometry identification of streptavidin-captured proteins from TgApiCox35-BioID and parental lines. Liquid chromatography–mass spectrometry analysis used an UltiMate 3000 RSLC system (Thermo Fisher Scientific) coupled in-line to an Orbitrap Fusion Lumos mass spectrometer (Thermo Fisher Scientific). Proteins were identified via alignment to a Toxoplasma proteome, and shown here are the proteins that were enriched in the SOD2-BioID sample.

ToxoDB GeneID	Protein Annotation	Protein Localization	Phenotype Score (Sidik et al., 2016 [6])	Human Homolog Similarity
TGME49_229920	Tg ApiCox35	Mitochondrion	−3.84	0.00%
TGME49_265370	Tg ApiCox16	Mitochondrion	1.56	0.00%
TGME49_221510	Tg ApiCox18	Mitochondrion	−3.28	0.00%
TGME49_247770	Tg ApiCox19	Mitochondrion	−2.61	0.00%
TGME49_286530	Tg ApiCox24	Mitochondrion	−2.82	0.00%
TGME49_264040	Tg ApiCox25	Mitochondrion	−2.54	0.00%
TGME49_306670	Tg ApiCox26	Mitochondrion	−3.68	0.00%
TGME49_297810	Tg ApiCox30	Mitochondrion	−3.64	0.00%
TGME49_209260	cytochrome c oxidase subunit 5b	Mitochondrion	−3.07	38.60%
TGME49_234420	ATPase, AAA family protein	Mitochondrion	−5.08	38.83%
TGME49_262640	Cg8 family protein	Mitochondrion	−3.49	0.00%
TGME49_210790	dihydroorotate dehydrogenase	Mitochondrion	−2.84	47.51%
TGME49_251780	heat shock protein	Mitochondrion	−5.09	66.67%
TGME49_257730	methionine aminopeptidase, type I	Mitochondrion	−3.51	42.96%
TGME49_236210	peptidase M16 family protein	Mitochondrion	−4.74	44.15%
TGME49_255910	PfMNL-2 CISD1 family iron-sulfur protein	Mitochondrion	1.64	48.72%
TGME49_207620	pyridine nucleotide-disulfide oxidoreductase domain-containing protein	Mitochondrion	−1.15	34.94%
TGME49_284580	ribose-phosphate diphosphokinase subfamily protein	Mitochondrion	−0.72	29.10%
TGME49_216530	ribosome recycling factor protein	Mitochondrion	−3.81	0.00%
TGME49_319730	YOU2 family C2C2 zinc finger protein	Mitochondrion	0.02	0.00%
TGME49_312160	hypothetical protein	Mitochondrion	−1.29	0.00%
TGME49_229420	cytochrome c	Cytoplasm	0.61	33.93%
TGME49_231350	glucosamine-fructose-6-phosphate aminotransferase	Plastid	−4.57	31.11%

## Data Availability

The original contributions presented in this study are included in the article/Supplementary Material. Further inquiries can be directed to the corresponding authors.

## Supplementary Materials

The following supporting information can be downloaded at: https://www.mdpi.com/article/10.3390/biom15070972/s1, Table S1: The parasite strains used; Table S2: qPCR primers; Table S3: The primers used for parasite genetic engineering; Table S4: The plasmids used.

## Abbreviations

The following abbreviations are used in this manuscript:

ROS	Reactive oxygen species
TgSOD2	*T. gondii* mitochondrial superoxide dismutase
mETC	Mitochondrial electron transport chain
HFFs	Human foreskin fibroblasts
mAID	Mini auxin inducible degron
TIR-1	Transport inhibitor response 1
BSO	Buthionine sulfoximine
PV	Parasitophorous vacuole
MMP	Mitochondrial membrane potential
SCF	Skp1-Cul1-F-box
IAA	Indoleacetic acid
TgSODs	*T. gondii* superoxide dismutases (1,2,3)
