# Supplementary material for: Elucidating the Role of Toxoplasma gondii’s Mitochondrial Superoxide Dismutase
Source: Biomolecules. 2025 Jul 7;15(7):972. doi: 10.3390/biom15070972 (PMC12292971; doi:10.3390/biom15070972)
Supplement: Supplementary file 1 [file biomolecules-15-00972-s001.zip › biomolecules-3698977-supplementary.pdf]

Strain	Use
RH	Type 1 strain used for initial qPCR surveys
K2 (ΔKu80ΔHXGPRT)	Parental strain for BioID tagged strains
TIR	Parental strain for mAID tagged strains
TgSOD2-BioID	Proximal biotinylation and TgSOD2 endogenous expression
TgApiCox35-BioID	Proximal biotinylation of TgApiCos35
TgSOD2-mAID	Inducible knockdown of TgSOD2
ER-mAID	Established mAID line, used for verification of inducible knockdown

Table S1: parasite strains used

Target	Gene ID	Primer Sequence
Actin F	TGGT1_209030	CACGAGAGAGGATACGGCTTC
Actin R	TGGT1_209030	CGATGTCGCTAGAGTCCTCAG
Catalase F	TGGT1_232250	CTCAGGTTCCGCCCGTAACATTC
Catalase R	TGGT1_232250	GCCAGCTTGTCGATGAGATGGAAG
thioredoxin reductase F	TGGT1_309730	CACAAACACGAACAACGGCG
thioredoxin reductase R	TGGT1_309730	GTGGACGGCTGAACAAAGTCG
superoxide dismutase 1 F	TGGT1_316310	GGAGACACTTCAGTTCCATC
superoxide dismutase 1 R	TGGT1_316310	GAAAATGGCTCCAGTAGACG
peroxiredoxin 2	TGGT1_266130	CATTACGAACGTCGCATCGC
peroxiredoxin 2	TGGT1_266130	CAGCGAGGAACGTCTTGATGTC
superoxide dismutase 2 F	TGGT1_316330	GGCACACCGTTCGCTGATAAG
superoxide dismutase 2 R	TGGT1_316330	CGTGGTTCCATGCTTGTGCTG
glutaredoxin F	TGGT1_227150	CTGGAGATTCTCAGGAACGC
glutaredoxin R	TGGT1_227150	GCGTGTAGTCCATCCGATC
glutathione reductase F	TGGT1_246920	CTGCGTGAAAAGCCAGATG
glutathione reductase R	TGGT1_246920	CGCAGTTGTCAAAGTCTGC
γ-glutamylcysteine synthetase F	TGGT1_232590	CGACCACGGTTCTTTACATG
γ-glutamylcysteine synthetase R	TGGT1_232590	GGTAGTCTGGTACTTTCCGTC

Table S2: qPCR primers

Number	Gene ID	Primer Sequence
1	TGGT1_316330	AAGTTGCGAAACAATCGTCTTGGTGG
2	TGGT1_316330	AAAACCACCAAGACGATTGTTTCGCA
3	TGGT1_316330	TTTCGCAGCTGAGAACTTGGTGAAAGCACTTGAAAGCAACT CTAAAATGGTGAGCAAGGG
4	TGGT1_316330	GACGCACAAATGACCATCGGCACTGATATAATTATGTTCCGA GAGGAAAACGAGAGACG
5	TGGT1_316330	TACTTCCAATCCAATTTACGAAGGTGAGGAGCATTCG
6	TGGT1_316330	TCCTCCACTTCCAATTTTAGCGTTGCTTTCAAGTGCTTTCAC C
7	TGGT1_316330	GCATTTGTGTCCTCCATCCGC
8	TGGT1_316330	CACGCCTCGGAGCATGTAAAG
9	TGGT1_316330	GAACCACCACATAACAGC
10	BioID_HA3x.LIC	CATAATCTGGAACATCGTAAGG
11	TGGT1_229920	AAGTTGAAGACGCTGTGGATCCAGTG
12	TGGT1_229920	AAAACACTGGATCCACAGCGTCTTCA
13	TGGT1_229920	ACACAAAATCAACTGGGTAGTGCCACGAGGCGACCTCGTTG CTAAAATTGGAAGTGGAGG
14	TGGT1_229920	GCGAAGGACGCCAAACGTTCATCGTTCCTCGTGTTTTGTTTC CTGCAAGTGCATAGAAG

Table S3: primers used for parasite genetic engineering

Plasmid	Use
SOD2-BirA-DHFR	Insertion of BioID tag onto TgSOD2
SOD2 gRNA	Expresses CAS9 and a TgSOD2 targeting gRNA
universal pU6	Bradley lab Cas9 expressing plasmid, gRNA are inserted into this plasmid
ApiCox35 gRNA	Expresses CAS9 and a TgApiCox35 targeting gRNA

Table S4: plasmids used
